# Persistent mTORC1 signaling in cell senescence results from defects in amino acid and growth factor sensing

**DOI:** 10.1083/jcb.201610113

**Published:** 2017-07-03

**Authors:** Bernadette Carroll, Glyn Nelson, Yoana Rabanal-Ruiz, Olena Kucheryavenko, Natasha A. Dunhill-Turner, Charlotte C. Chesterman, Qabil Zahari, Tong Zhang, Sarah E. Conduit, Christina A. Mitchell, Oliver D.K. Maddocks, Penny Lovat, Thomas von Zglinicki, Viktor I. Korolchuk

**Affiliations:** 1 Institute for Cell and Molecular Biosciences, Newcastle University, Newcastle upon Tyne, UK; 2 Institute of Cellular Medicine, Newcastle University, Newcastle upon Tyne, UK; 3 Wolfson Wohl Cancer Research Centre, Institute of Cancer Sciences, University of Glasgow, Glasgow, Scotland, UK; 4 Cancer Program, Monash Biomedicine Discovery Institute, Department of Biochemistry and Molecular Biology, Monash University, Clayton, Victoria, Australia

## Abstract

Mammalian target of rapamycin complex 1 (mTORC1) and cell senescence are intimately linked to each other and to organismal aging. Inhibition of mTORC1 is the best-known intervention to extend lifespan, and recent evidence suggests that clearance of senescent cells can also improve health and lifespan. Enhanced mTORC1 activity drives characteristic phenotypes of senescence, although the underlying mechanisms responsible for increased activity are not well understood. We have identified that in human fibroblasts rendered senescent by stress, replicative exhaustion, or oncogene activation, mTORC1 is constitutively active and resistant to serum and amino acid starvation. This is driven in part by depolarization of senescent cell plasma membrane, which leads to primary cilia defects and a resultant failure to inhibit growth factor signaling. Further, increased autophagy and high levels of intracellular amino acids may act to support mTORC1 activity in starvation conditions. Interventions to correct these phenotypes restore sensitivity to the mTORC1 signaling pathway and cause death, indicating that persistent signaling supports senescent cell survival.

## Abstract (precis)

It is unclear how mTORC1 signaling differs in senescent and young cells. Carroll et al. show that senescence leads to constitutive mTORC1 activation and resistance to serum and amino acid starvation. This is associated with elevated autophagy, depolarization of cell plasma membrane, and primary cilia defects.

## Introduction

Cellular senescence is an irreversible cell cycle exit that is a key tumor suppressor mechanism and also directly contributes to aging (López-Otín et al., 2013). Indeed, clearance of senescent cells can improve aging phenotypes (Baker et al., 2011, 2016). Senescence is characterized by proliferation arrest, increase in cell size and mitochondrial mass together with mitochondrial dysfunction, and increased secretion of proinflammatory and pro-oxidant signals (Passos et al., 2007, 2010; Rodier et al., 2009; López-Otín et al., 2013). This increase in cell growth and metabolism is supported in part by mTORC1 (Zhang et al., 2000; Demidenko and Blagosklonny, 2008; Carroll et al., 2013; Xu et al., 2013; Herranz et al., 2015; Correia-Melo et al., 2016), a conserved serine/threonine kinase that specifically regulates protein translation and nucleotide and lipid biogenesis and inhibits the catabolic process of autophagy (Laplante and Sabatini, 2012; Carroll et al., 2015).

Amino acids are necessary and sufficient for mTORC1 activation, the magnitude of which is greatly enhanced in the presence of growth factors (Hara et al., 1998; Long et al., 2005; Carroll et al., 2016). Growth factors signal via phosphoinositide 3-kinase (PI3K)/Akt and tuberous sclerosis complex (TSC1/2) to activate the small GTPase Rheb, which is the master activator of mTORC1 (Dibble and Cantley, 2015). TSC2 localization to the lysosome, and therefore Rheb activity, is controlled by availability of growth factors and amino acids, specifically arginine, (Demetriades et al., 2014; Menon et al., 2014; Carroll et al., 2016). Amino acids further regulate mTORC1 activity by controlling its localization at the lysosome via the signaling cascade upstream of Ragulator complex and Rag GTPases (Laplante and Sabatini, 2012). Starvation of growth factors or amino acids inhibits mTORC1 and activates autophagy. Autophagy involves the engulfment of cytoplasmic contents into double membrane–bound vesicles called autophagosomes, which fuse with lysosomes, degrading their contents, which are subsequently released into the cytoplasm (Carroll et al., 2015). Starvation therefore shifts the cell from an anabolic to a catabolic program to liberate nutrients and ensure cell survival.

mTORC1 activity promotes senescence phenotypes; however, it is unclear how mTORC1 signaling differs in senescent versus young cells. Indeed, its activity appears to be only moderately elevated in senescence (Demidenko and Blagosklonny, 2008; Dalle Pezze et al., 2014; Correia-Melo et al., 2016), although it has been reported to become insensitive to serum in senescent cells (Zhang et al., 2000). To further understand the underlying mechanisms by which mTORC1 is dysregulated in senescence, we investigated the ability of mTORC1 and autophagy to sense and appropriately respond to changes in extracellular nutrient availability in young and senescent cells.

## Results and discussion

**Figure 1.**
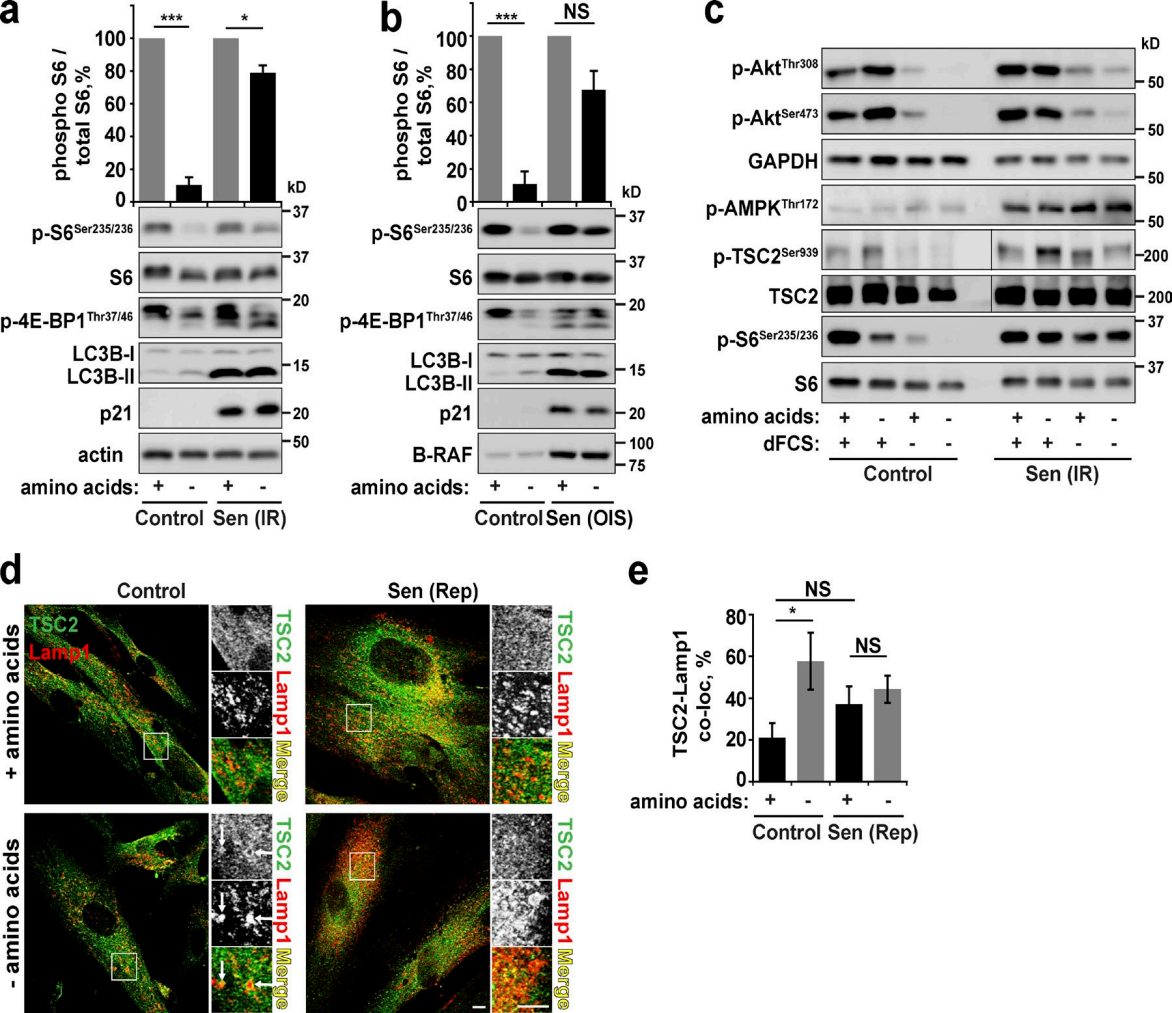
**PI3K/Akt/mTOR/autophagy are insensitive to starvation in senescent cells.** (a) Immunoblot analysis of mTORC1 and autophagy activity in control and senescent (Sen(IR); 30 d after 20 Gy x-ray irradiation) primary human fibroblasts after overnight serum starvation with or without 1-h amino acid starvation. (b) As in panel a, but senescence was achieved by transduction of cells with oncogenic mutant B-RAF^V600E^ (oncogene-induced senescence, OIS). (c) Immunoblot analysis of signaling cascades upstream of mTORC1 after starvation protocols as described in panel a. (d and e) Proliferating or replicative senescent (Sen(Rep)) cells were treated as in a, fixed, and immunostained for antibodies against TSC2 and Lamp1. White arrows indicate recruitment of TSC2 to lysosomes (d). Colocalization between Lamp1 and TSC2 (e) was analyzed. Error bars represent SEM; all experiments, *n* = 3 (for colocalization, at least 10 cells imaged per experimental repeat). Student’s *t* test performed between groups: *, P < 0.05; ***, P < 0.001; NS, not significant. Bars, 10 µm.

Upon removal of serum and amino acids, proliferating primary human fibroblasts (control) show a significant decrease in mTORC1 signaling (phospho S6 and 4EBP1) and a concomitant increase in LC3B-II levels, a marker for autophagy (Fig. 1, a and b). In contrast, mTORC1 activity persists in the absence of these mitogenic signals in stress-induced senescent (20 Gy irradiation), oncogene-induced senescent (B-RAF^V600E^ transduction), and replicative senescent cells (Fig. 1, a and b; and Fig. S1 a). This is accompanied by a lack of increase in LC3-II levels, although interestingly, the basal levels of LC3B-II are significantly higher in senescent cells than in control cells (Narita et al., 2011). We confirmed that this phenotype is specific to senescence and is not simply a result of cell cycle exit (quiescence; Fig. S1 a; Demidenko and Blagosklonny, 2008). Different starvation methods indicate that signaling pathways dependent on both growth factors (Zhang et al., 2000) and amino acids are perturbed in senescent cells. Indeed, we observed higher levels of phosphorylated Akt and TSC during starvation (but no changes in AMPK) in addition to the insensitivity of phospho-S6 to any starvation protocol (Fig. 1 c). Furthermore, the localization of TSC2 to the lysosomes, which we have shown previously to be regulated by both growth factors and amino acids (Carroll et al., 2016), is perturbed in senescence (Fig. 1, d and e). This perturbation directly, but partially, contributes to dysregulated mTORC1, as overexpression of TSC2 reduces mTORC1 activity and restores some sensitivity to amino acid starvation (Fig. S1 b). In senescent cells, mTOR localization remains sensitive to starvation by relocalizing to the cytoplasm; however, note that after starvation, mTOR-Lamp1 colocalization levels remain higher than in control cells, which may contribute to increased mTORC1 activity (Narita et al., 2011; Fig. S1 c). Together, these data indicate that mTORC1 activity in senescent cells becomes constitutive and insensitive to external and internal mitogenic cues; in agreement with previous studies, this includes growth factors (Zhang et al., 2000), but we also demonstrate that senescent cells are insensitive to amino acid availability. The activity of mTORC1, however, is not necessarily grossly elevated compared with proliferating, young cells.

**Figure 2.**
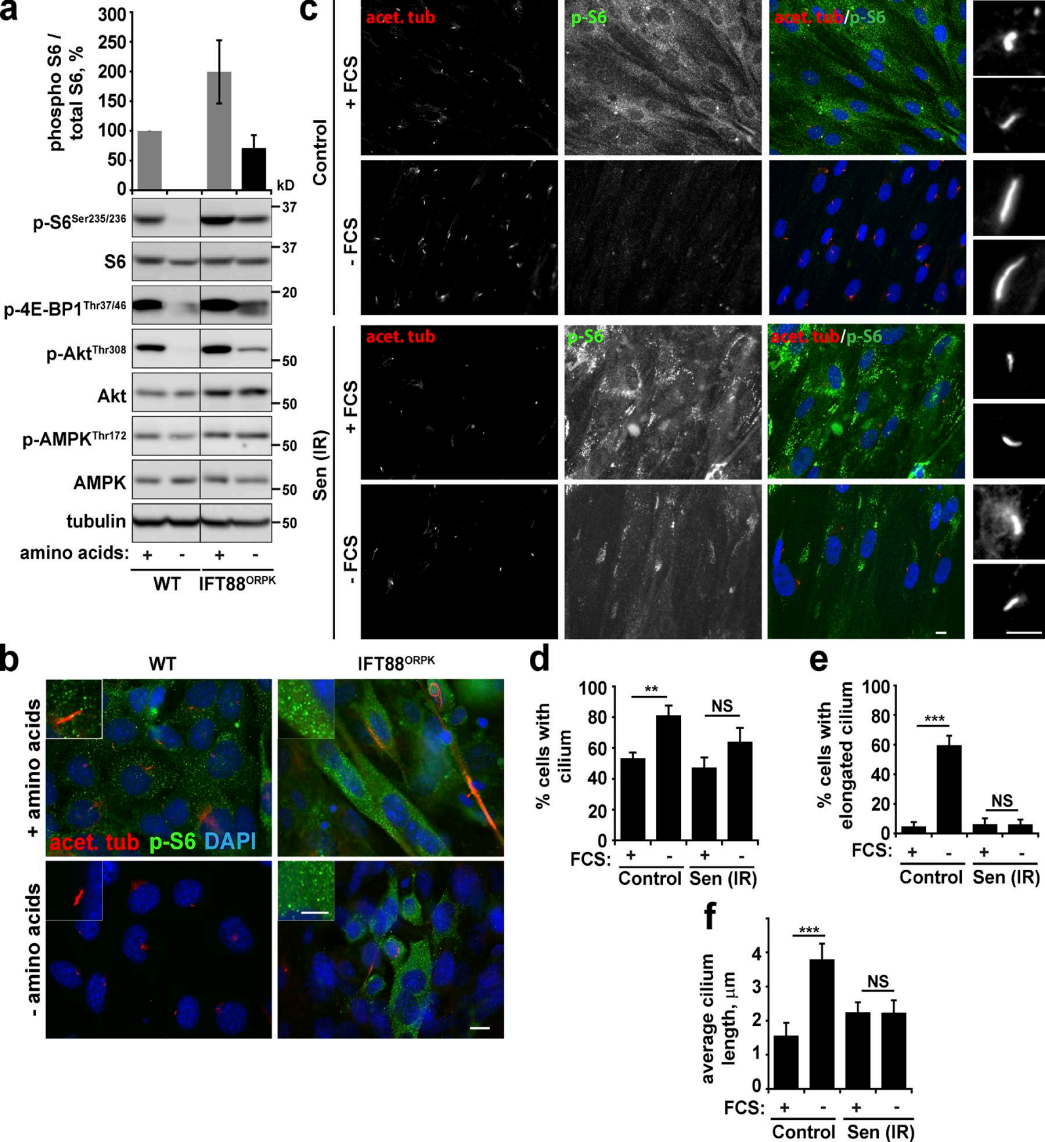
**Senescent cells fail to induce primary cilium elongation.** (a and b) Wild-type and IFT88^ORPK^ chondrocytes (which are deficient in primary cilia; Wann et al., 2012) were grown until confluent and subjected to serum starvation overnight. Cells were either lysed and subjected to Western blotting (a) or fixed and immunostained with antibodies against acetylated tubulin and phospho-S6 (b). (c–f) Confluent control and senescent (20 Gy irradiation; Sen (IR)) fibroblasts were serum-starved overnight (–FCS), fixed, and immunostained for acetylated tubulin and phospho-S6 (c). Percentage of cells with primary cilium (d), percentage of cells with elongated primary cilium (e), and mean cilium length (f) were quantified. Error bars represent SEM; all experiments, *n* = 3 (for immunofluorescence, at least five fields of view imaged per experimental repeat). Student’s *t* test performed between groups: **, P < 0.01; ***, P < 0.001; NS, not significant. Bars: (main) 10 µm; (insets) 5 µm.

Previous studies indicated that deficiencies in primary cilia formation are associated with enhanced PI3K/mTORC1 signaling (Boehlke et al., 2010; Wang et al., 2015). We initially confirmed that indeed, Akt and mTORC1 signaling is enhanced in cells deficient in cilia formation (chondrocytes harboring an IFT88 hypomorphic mutation; Fig. 2, a and b). Importantly, similar to senescent cells, cilia-deficient cells are resistant to starvation. Akt and mTORC1 signaling persisted in the absence of serum and amino acids, and similar defects in starvation-dependent TSC2 localization to lysosomes were observed (as well as the trend for higher levels of mTOR-Lamp1 colocalization after starvation; Fig. 2, a and b; and Fig. S1, d and e). It is interesting to note that the similarities between senescent and cilia-deficient cells extend to the observed increase in lysosomal content (Fig. S1, c–e). Whether this is a result of defective mTORC1 signaling—for example, through perturbed TFEB/TFE3-dependent transcriptional regulation of lysosomal proteins (Napolitano and Ballabio, 2016)—or a result of defective lysosomal turnover remains to be investigated. At the same time, we noted that upon starvation of HeLa cells, patches of phospho-S6–positive cells were still observed in the confluent monolayer, which correlates with a consistent failure of these cells to grow primary cilia (Fig. S1 f). Together, these data indicate that PI3K/mTORC1 activity is negatively regulated by primary cilia, and signaling cannot be efficiently inhibited in their absence. Therefore, we asked whether a lack of cilia formation could represent the underlying mechanism of constitutive mTORC1 activity in senescence. Indeed, although serum starvation of confluent fibroblasts induced significant elongation of primary cilia, cilia present in senescent cells failed to elongate upon starvation (Fig. 2, c–f), even though the same number of short cilia were observed in resting conditions (Fig. 2, c and d).

**Figure 3.**
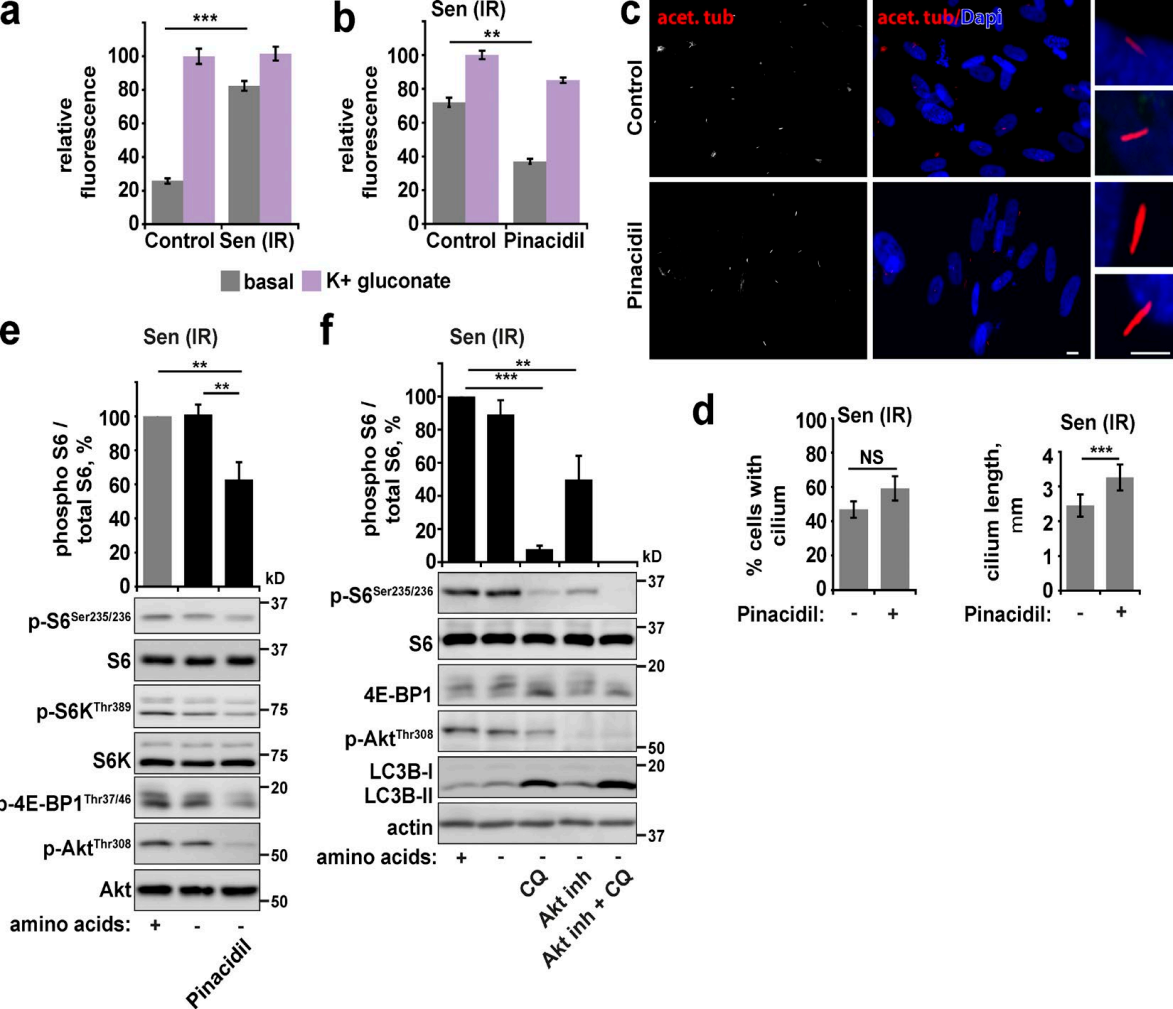
**Restoration of membrane potential in senescent cells promotes cilia elongation.** (a) Resting and maximum membrane potential (maximum achieved by incubation for 5 min with 80 mM potassium gluconate) were measured in control and senescent (20 Gy irradiation; Sen (IR)) fibroblasts using DiSBAC_2_(3) dye. Depolarization is indicated by increased fluorescence. Data are represented relative to maximum. (b) Senescent cells treated as in a but in the presence or absence of 100 µM pinacidil overnight. (c and d) Senescent fibroblasts were treated with pinacidil overnight in serum-free medium. Cells were fixed and stained for acetylated tubulin (c). Percentage of cells with cilia and cilia length were quantified (d). (e) Senescent fibroblasts were starved of serum overnight and amino acids for 1 h in the presence or absence of pinacidil as indicated, lysed, and analyzed by immunoblot for mTORC1 activity. (f) Senescent fibroblasts were starved of serum overnight and amino acids for 1 h in the presence or absence of 50 µM autophagy inhibitor chloroquine (CQ), 10 µM Akt inhibitor, or both inhibitors as indicated. Cells were lysed and immunoblotted. Error bars represent SEM; all experiments, *n* = 3 (for immunofluorescence, at least five fields of view imaged per experimental repeat). Student’s *t* test performed between groups: **, P < 0.01; ***, P < 0.001; NS, not significant. Bars: (main) 10 µm; (insets) 5 µm.

Defects in cilia formation could result from differences in the properties of the plasma membrane in senescent cells. Indeed, we found that the plasma membrane becomes depolarized upon senescence acquisition (Fig. 3 a). Interestingly, membrane depolarization is clearly linked to the acquisition of senescence, as long-term culture of fibroblasts in the presence of pinacidil (which can partially prevent membrane depolarization in senescent cells) increases the rate of proliferation and postpones the onset of growth arrest (Fig. 3 b and Fig. S2, a and b). Importantly, restoration of senescent fibroblast plasma membrane potential with pinacidil treatment not only rescued the primary cilia elongation defect of senescent cells (Fig. 3, c and d) but also promoted complete starvation-dependent inhibition of Akt and, to a lesser extent, mTORC1 signaling (Fig. 3 e). Interestingly, pinacidil was also able to induce a small but significant increase in cilia length and concomitant significant decrease in phosphorylation of Akt and S6 in starved control fibroblasts (Fig. S2, c and d). Mechanistically, the effect of pinacidil on signaling is likely to be upstream or via the observed cilia elongation, as pinacidil does not significantly reduce signaling in cilia-deficient cells and does not rescue the elevated signaling we previously observed in these cells in starved conditions (Fig. S2 e). Rather, pinacidil in fact increased phosphorylation of S6 in cilia-deficient cells in starvation conditions. Together, these data highlight the central importance of membrane potential in responding appropriately to mitogenic signals and suggest that membrane depolarization, which is acquired during senescence, leads to defects in cilia formation, which ultimately causes insensitivity of intracellular signaling pathways to nutrient availability.

Although the sensitivity of growth factor signaling was restored by hyperpolarization of the membrane, residual mTORC1 signaling was still observed (Fig. 3 e). Because cilia are not known to affect amino acid sensing, we hypothesized that additional perturbation of amino acid sensing may further contribute to the mTORC1 phenotype. We investigated the contribution of autophagy for three main reasons. First, it is a major catabolic process that generates free amino acids in the cytoplasm (Carroll et al., 2015); indeed, in normal cells, prolonged periods of starvation can lead to the reactivation of mTORC1 as a result of autophagy-derived liberation of free amino acids (Yu et al., 2010). Second, consistent with previous data (Narita et al., 2011; Dalle Pezze et al., 2014), basal autophagy levels are significantly higher in senescent cells, and the flux through the pathway is preserved (Fig. 1, a and b; and Fig. S2 f). Third, the amount of free amino acids per cell is increased in both fed and starved senescent compared with control cells, potentially resulting in their concentrations being sufficient for mTORC1 activation at localized sites, e.g., lysosomes, even during starvation (although the levels are similar when normalized to protein levels; Fig. S2, g and h). We therefore hypothesized that in senescent cells, elevated autophagy generates intracellular amino acids that can support mTORC1 activity even on removal of exogenous amino acids. To test this, we inhibited autophagy using chloroquine (to inhibit autophagosome degradation) or *Atg5* siRNA (to suppress autophagosome formation). Chloroquine significantly restored amino acid starvation–dependent inhibition of mTORC1 (Fig. 3 f), whereas partial rescue was observed after *Atg5* siRNA treatment, corresponding to partial knock-down efficiency achieved in senescent cells (Fig. S2 i). In agreement with the role of persistent growth factor signaling in mTORC1 phenotype in senescent cells, treatment of cells with an Akt inhibitor also partially restored the mTORC1 phenotype, whereas treatment with an Akt inhibitor and chloroquine completely abolished mTORC1 signaling in starved cells (Fig. 3 f). Importantly, the ability of chloroquine to restore signaling sensitivity to senescent cells is not a result of general mTORC1 inhibition, as it did not inhibit Akt or S6 phosphorylation in control, nonstarved cells (Fig. S2 j). Furthermore, chloroquine had no effect on membrane potential or cilium length in senescent cells, supporting a mode of action more proximal to mTORC1, i.e., modulating autophagy/amino acids (Fig. S2, k and l). Collectively, these data support the conclusion that constitutive mTORC1 activity in senescent cells is supported by multiple independent pathways including cilia defects and high levels of autophagy.

**Figure 4.**
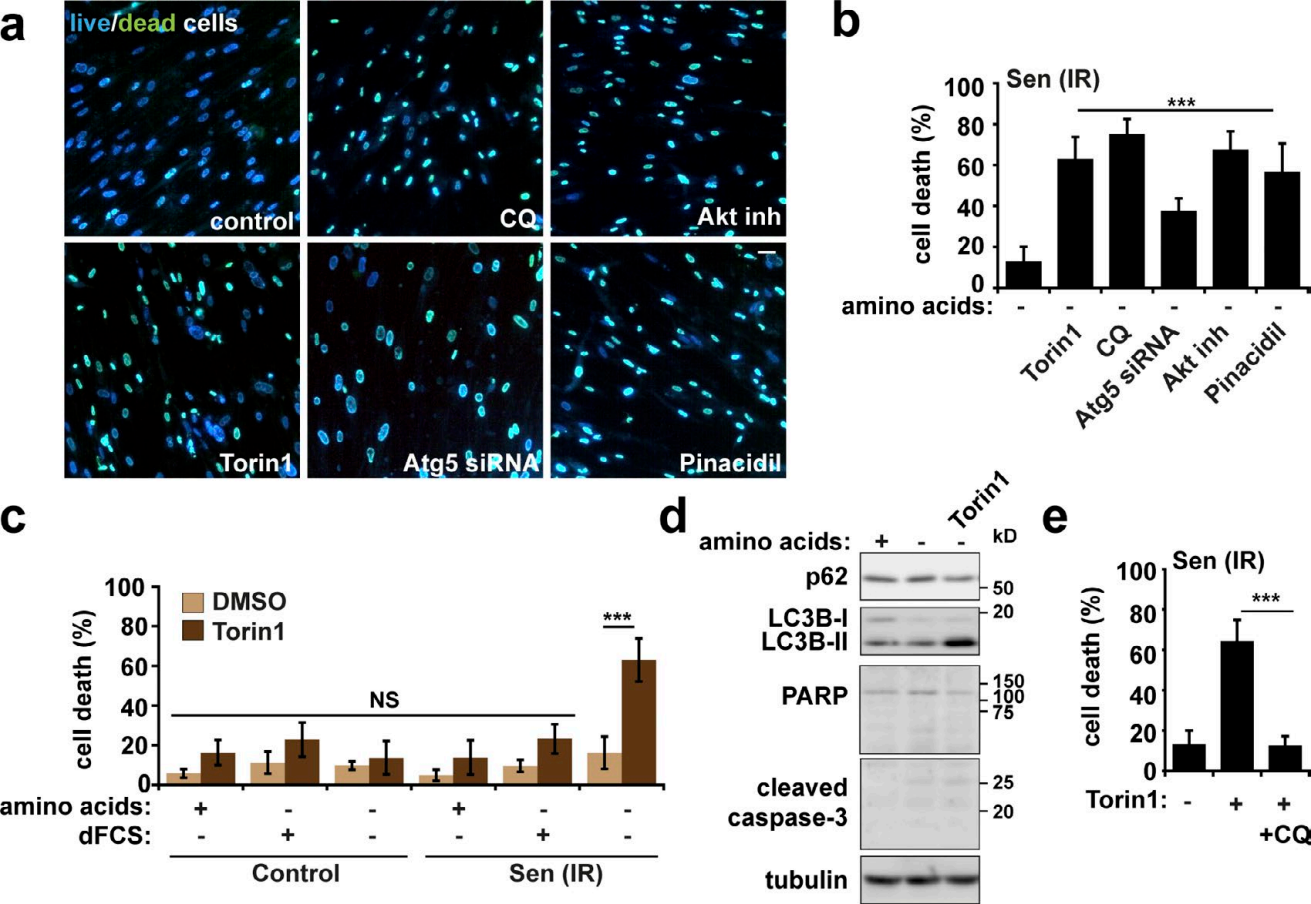
**Persistent Akt/mTORC1 signaling supports senescent cell survival and can be exploited to specifically promote cell death.** (a and b) Senescent fibroblasts were starved of serum and amino acids overnight in the presence or absence of the mTOR kinase inhibitor 200 nM Torin1 (overnight), 50 µM autophagy inhibitor chloroquine (CQ; 4 h), or 10 µM Akt inhibitor (4 h) as indicated. Cells were incubated with fluorescent ReadyProbe Cell Viability reagents (a), and percentage cell death was quantified (b). (c) Control or senescent (20 Gy irradiation; Sen(IR)) fibroblasts were starved of serum and amino acids in the presence or absence of 200 nM Torin1 for 24 h. Cells were incubated with fluorescent ReadyProbe Cell Viability reagents, and cell viability was quantified (c). (d) Senescent fibroblasts were starved in the presence or absence of Torin1. Cells were lysed and analyzed for markers of apoptosis (PARP and cleaved caspase-3) and autophagy (p62 and LC3). (e) Senescent fibroblasts were starved in the presence or absence of Torin1 and chloroquine as indicated. Percentage cell death was analyzed by ReadyProbe cell viability reagents. Error bars represent SEM; for all experiments, *n* = 3 (for immunofluorescence, at least five fields of view imaged per experimental repeat). Student’s *t* test performed between groups: ***, P < 0.001; NS, not significant. Bar, 30 µm.

Next, we investigated whether senescent cells are dependent on persistent mTORC1 activity for their survival in starvation conditions. To test this, we used Torin1, chloroquine, Akt inhibitor, and pinacidil to suppress persistent mTORC1 in starved senescent cells (Fig. 3, e and f) and measured cell survival (Fig. 4, a and b; and Fig. S3 a). Indeed, compared with starvation alone, all of these mTORC1-inhibiting drugs induced significant senescent cell death (Fig. 4, a and b; and Fig. S3 a). Importantly, we confirmed that only minimal cell death occurred in control fibroblasts when starved in the presence of Torin1 for 24 h (Figs. 4 c and S3 b). Furthermore, only in the absence of serum and amino acids was significant cell death induced in senescent cells (Figs. 4 c and S3 b). Further evidence that persistent mTORC1 activity represents survival vulnerability comes from the fact that starvation plus Torin1 selectively kills nonsenescent *TSC2^−/−^* mouse embryonic fibroblasts (MEFs, which have constitutively active Rheb, and therefore mTORC1) compared with wild-type controls (Fig. S3 c). Consistent with resistance of senescent cells to apoptosis (Wang, 1995), we found no evidence of apoptotic cell death in starved senescent cells treated with Torin1. Instead, we found a further induction of autophagy over and above the increased autophagy levels in senescent cells at basal conditions (Fig. 4 d). Inhibition of autophagy by chloroquine 1 h before the observed cell death rescued Torin1-induced senescent cell death (Fig. 4 e). Together, these observations indicate that increased autophagy contributes to mTORC1 deregulation (Narita et al., 2011) and survival of senescent cells during starvation, whereas persistent mTORC1 prevents further hyperactivation of autophagy, which could lead to cell death (Shimizu et al., 2014).

In summary, we have identified that extensive rewiring occurs during the acquisition of senescence (Dalle Pezze et al., 2014), leading to the constitutive activation of mTORC1, which is resistant to both serum and amino acid starvation. Although mTORC1 activity appears to be supported by autophagy upon starvation, evidence suggests that persistent mTORC1 simultaneously prevents senescent cells from realizing their full autophagic potential, which would otherwise lead to cell death. This seemingly contradictory role for autophagy as a prosurvival and cell death mechanism is a phenomenon also shown to contribute to tumorigenesis and neurodegeneration (Levine and Kroemer, 2008).

In addition to increased autophagy, we identified that constitutive mTORC1 is supported by defects in sensing growth factor starvation. Specifically, senescent cells show defects in starvation-induced primary cilia growth (Bishop et al., 2010; Breslin et al., 2014) and, as a consequence, fail to efficiently suppress Akt and mTORC1 upon serum starvation (Boehlke et al., 2010; Wang et al., 2015). For the first time, we have shown that low plasma membrane potential supports these prosurvival signaling changes in senescence, restoration of which promoted starvation-induced cilia growth and thus Akt and mTORC1 inhibition. Despite these new insights, further work is required to fully realize the signaling and metabolic consequences of perturbed membrane potential in senescent cells, the underlying mechanisms that lead to this perturbation, and its relationship with other senescence-associated changes such as disrupted mitochondrial membrane potential (Miwa et al., 2014; Ziegler et al., 2015). It also remains unclear how membrane potential supports cilia growth and the mechanisms by which elongated cilia affect growth factor signaling. Exploring these causal and consequential interactions will undoubtedly improve our understanding of basic cell biology of senescence and in the wider areas of aging and ciliopathies.

Overall, our findings expand on the understanding of how mTORC1 is dysregulated in senescence and describe the underlying specific mechanisms of dysregulation, which may be targetable for senolytic interventions. This growing understanding has important implications for future health and lifespan interventions.

## Materials and methods

All chemicals were from Sigma-Aldrich unless indicated otherwise.

### Cells and drug treatments

HeLa cells, primary human fibroblasts (MRC5), human diploid fibroblasts, TSC2-deficient (*TSC2^−/−^*) and wild-type (*TSC2^+/+^*) MEFs (gift from D. Kwiatkowski, Harvard University, Cambridge, MA), and IFT88^ORPK^ and control chondrocytes (gift from M. Knight, Queen Mary University of London, UK; see Wann et al., 2012, for specific culture and differentiation protocols) were cultured in DMEM (D6546) with 2 mM l-glutamine, 10% FBS, and 100 U/ml penicillin-streptomycin at 37°C, 5% CO_2_. In brief, *TSC2^−/−^* MEFs were produced from TSC2^+/−^ or TSC2^+/−^TP53^−/−^ intercrosses (Zhang et al., 2003). Note these cells are TP53^−/−^. The original TSC2^+/−^ MEFs were produced by electroporating a TSC2 targeting construct into J1 embryonic stem cells. After confirmation of homologous recombination, successful clones were used to impregnate female mice (Onda et al., 1999). HeLa cells were incubated with 100 nM rapamycin overnight in serum-free media to monitor the effect of mTORC1 inhibition on cilia formation. Human fibroblasts were incubated with pinacidil (100 µM), Torin1 (200 nM; Tocris Bioscience), chloroquine (50 µM), and Akt inhibitor (1708-1; BioVisions; 10 µM for 1 or 4 h, as indicated).

### Senescence induction

Stress-induced senescence was achieved by exposing cells to 20 Gy irradiation using an X-rad225 irradiator (Precision X-ray, Inc.). Immediately after irradiation, medium was refreshed and was changed every 3 or 4 d for at least 30 d. Oncogene-induced senescence was induced by viral transduction of mutant B-RAF^V600E^ (gift from D. Peeper, The Netherlands Cancer Institute, Amsterdam, Netherlands). Experiments were performed 10 d after transduction. Replicative senescence was achieved via replication exhaustion and defined by less than 0.5 population doublings for at least 4 wks.

### siRNA

ON-TARGETplus SMARTpool siRNA against human Atg5 (L-004374-00) and nontargeting SMARTpool siRNA (D-001810-10) were purchased from GE Healthcare. Final siRNA concentrations of 100 nM were used for 96 h for silencing, and transfections were performed using DharmaFECT 2 (GE Healthcare) as per company instructions.

### TSC2 Transduction

Overexpression of wild-type TSC2 was performed by lentiviral transduction. The pLKO.1 WT TSC2 construct was a gift from B. Manning (Harvard School of Public Health, Cambridge, MA; Menon et al., 2014). In brief, viruses were produced by the following protocol. 6 × 10^6^ HEK293FT cells were seeded 24 h before transfection in 10-cm dishes. Cells were transfected with control or WT TSC2 plasmid (5 µg) along with the packaging plasmids, VSV-G (2.5 µg), and Δ8.9 (3.75 µg) using Lipofectamine 2000 as per company instructions. Viruses were harvested 48 h after transfection, filtered, and incubated with senescent fibroblasts overnight. The virus-containing medium was replaced the next morning, and cells were left for a further 3 d before experiments.

### Starvation and recovery protocols

Unless otherwise stated, serum starvation was performed overnight (18 h), and amino acid starvation (including in the presence of dialyzed FCS) was performed for 1 h (after one wash with PBS). Amino acid starvation was performed using RPMI without amino acids (US Biologicals). For cell death assays, cells were starved of serum and amino acids for 24 h. FCS was dialyzed with cassettes of <2 kD cutoff as per company instructions (Thermo Fisher Scientific).

### Immunoblotting

Immunoblotting was performed as described previously (Korolchuk et al., 2011; Carroll et al., 2016). In brief, cells were lysed in RIPA buffer (50 mM Tris-HCl, pH 7.4, 150 mM NaCl, 1% NP-40, 0.5% sodium deoxycholate, and 0.1% SDS, supplemented with Halt protease and phosphatase inhibitors; 1861280; Thermo Fisher Scientific) on ice. Protein concentrations of lysates were measured using DC protein assay (500-0112; Bio-Rad Laboratories), and equal amounts of protein (20–40 µg) were subjected to SDS-PAGE and immunoblotted. The following primary antibodies were used: rabbit anti–phospho S6^Ser235/236^ (4856, 1:2,000), rabbit anti-S6 (2217, 1:2,000), rabbit anti–phospho Akt^Ser473^ (9271, 1:1,000), rabbit anti–phospho Akt^Thr308^ (4056, 1:1,000), Akt (9272, 1:1,000), rabbit anti–phospho 4E-BP1^Thr37/46^ (2855, 1:2,000), rabbit anti–phospho AMPK^Thr172^ (2535, 1:1,000), rabbit anti–phospho TSC2^Ser939^ (3615S, 1:500), rabbit anti-p21 (2947, 1:1,000), rabbit anti-LC3B (3868, 1:2,000), rabbit anti-PARP (9532, 1:1,000), and rabbit anti–cleaved caspase-3^Asp175^ (9661, 1:1,000), all purchased from Cell Signaling Technology. Other antibodies used in this study include mouse anti–α-tubulin (12G10, 1:10,000; Developmental Studies Hybridoma Bank), B-RAF (sc-5284, 1:2,000; Santa Cruz Biotechnology, Inc.), rabbit anti-Atg5 (A0856, 1:1,000; Sigma-Aldrich). Secondary antibodies conjugated to HRP were all used at 1:5,000 for 1 h at RT. Clarity western ECL substrate (Bio-Rad Laboratories) was used to visualize chemiluminescence on LAS4000 (Fujifilm). Quantification of blots was performed using ImageJ v. 1.41 (NIH).

### Immunofluorescence

Immunofluorescence was performed essentially as described previously (Korolchuk et al., 2011; Carroll et al., 2016). In brief, cells were fixed in 4% formaldehyde in PBS for 10 min, permeabilized with 0.5% Triton X-100 for 10 min, and blocked in normal goat serum/PBS-Tween for 1 h, all at RT. The following primary antibodies were used: rabbit anti-mTOR (2972, 1:200; Cell Signaling Technology), rabbit anti-TSC2 (4308, 1:1,000; Cell Signaling Technology), mouse anti-Lamp1 (for human cells, 1:1,000; Abcam), rat anti-Lamp1 (for mouse cells, 1:1,000; Developmental Studies Hybridoma Bank), mouse anti–acetylated tubulin (T7451, 1:1,000; Sigma-Aldrich), and rabbit anti–phospho S6^Ser235/236^ (#4856, 1:200; Cell Signaling Technology). Cells were washed and incubated with the appropriate secondary antibodies (1:1,000; Thermo Fisher Scientific) for 1 h at RT.

Plasma membrane potential was measured by live imaging confocal microscopy. Confocal images were collected on either an LSM 510 META Confocal Microscope (ZEISS) or an SP8 (Leica Biosystems) using a 63× Plan-Apo/1.4-NA oil objective, at 37°C, 5% CO_2_. Proliferating and senescent fibroblasts were incubated with DMEM containing 0.5 µM DiSBAC_2_(3) (Thermo Fisher Scientific) in the presence or absence of pinacidil overnight. Images were acquired with either LSM v. 3.2 (ZEISS) or LasX v. 2.9 (Leica Biosystems) using glass-bottom dishes (Willco) with cells in medium. The relative fluorescence from DisBAC_2_(3) (red emission, captured using a 560- to 580-nm bandpass filter) increases as the cells take up more dye, relative to their membrane potential. Maximum depolarization was achieved by incubation with 80 mM potassium gluconate for 5 min. The resting membrane potential was normalized to this value. For immunofluorescence, coverslips were mounted on slides with Prolong Gold antifade reagent with DAPI (Thermo Fisher Scientific). Secondary antibodies were conjugated with Alexa Fluor 488 or 594. Images were captured using the Leica SP8 as described, with samples at RT. All analyses were performed in ImageJ using regions of interest to determine cell intensity in the respective fluorescent channels. For DisBAC_2_(3) analysis, *z*-stack images containing the whole volume of the cell were flattened into maximum projections before analysis. For colocalization, *z*-stack images containing the whole volume of the cell were analyzed by colocalization plugin after a threshold was applied. For presentation purposes, stacks were *z*-projected to maximum intensity. Cell viability was assessed using Ready Probes (Thermo Fisher Scientific) as per company instructions. Cells were imaged on a DM-IL inverted fluorescence microscope (Leica Biosystems) equipped with an Invenio 3SII digital camera (3.0 Mpix Color CMOS; Indigo Scientific).

### Liquid chromatography-mass spectrometry

HeLa cells were seeded (in triplicate) in six-well plates and cultured in standard DMEM until 90% confluent. Cells were treated as indicated. Cells were washed once with cold PBS and lysed (50% methanol/30% acetonitrile/20% dH_2_O) at a concentration of 2 × 10^6^ cells/ml. Samples were vortexed for 45 s and centrifuged at 13,000 rpm. Cleared lysates were subjected to liquid chromatography-mass spectrometry (LC-MS) as follows, using a three-point calibration curve with universally labeled carbon-13/nitrogen-15 amino acids for quantification. Cell lysate (10 µl) solution was injected into an Accela 600 LC system coupled to an Exactive mass spectrometer (Thermo Fisher Scientific). Chromatographic separation was performed on a Sequant ZIC-HILIC column (150 × 4.6 mm, 3.5 µm; Merck) with mobile phase A (water) and B (acetonitrile) both consisting of 0.1% formic acid (vol/vol) at the flow rate of 0.3 ml/min. A gradient elution program was used: mobile phase A increasing from 20 to 80% in 30 min and holding A at 92% for 5 min, followed by 10-min reequilibration with 20% A. The Exactive mass spectrometer was equipped with a heated electrospray ionization source and operated in an electrospray ionization–positive and –negative switching mode with a scan range of 70–1200 *m*/*z* at a resolution of 50,000. The obtained LC-MS raw data were converted into .mzML files with ProteoWizard and imported into MZMine 2.10 to conduct peak extraction, sample alignment, and metabolite identification.

### Quantification and statistical analysis

Quantification of confocal images was performed using the colocalization plugin in ImageJ to measure the colocalization between mTOR or TSC2 and the lysosomal protein Lamp1. A constant threshold was applied to all the images in the *z*-stack and for every image within each experiment. After application of the colocalization plugin, all channels were projected (maximum) and quantified using Analyze particle plugin (particles 5 pixels and larger were included). The data were expressed as a percentage of mTOR or TSC2 that colocalized with Lamp1. Quantification was performed on 20–40 cells per condition in three independent experiments. Cilia were quantified using three different methods: first, the percentage of cells with cilia were manually counted; second, the percentage of cells with elongated cilia were manually counted (based on a threshold set by the counter); and third, the mean cilium length was determined (measured via ImageJ). All scoring and quantification were performed blinded, and >100 cells were counted per slide. Quantification is based on at least three independent experiments unless otherwise stated. Quantification of immunoblots was performed using ImageJ. Two-tailed, unpaired Student’s *t* tests were performed on experimental data from at least three individual experiments.

### Online supplemental material

Fig. S1 shows that resistance of PI3K/Akt/mTOR signaling occurs in senescent and cilia-deficient cells possibly via perturbation of TSC2 and mTOR localization. Fig. S2 shows that changes in plasma membrane potential and autophagy in senescence support persistent mTORC1 signaling. Fig. S3 shows targeting of persistent mTORC1 signaling to promote senescent cell death.

## Supplementary Material

Supplemental Materials (PDF)
