# Supplementary material for: Persistent mTORC1 signaling in cell senescence results from defects in amino acid and growth factor sensing
Source: J Cell Biol. 2017 Jul 3;216(7):1949–57. doi: 10.1083/jcb.201610113 (PMC5496614; doi:10.1083/jcb.201610113)
Supplement: Supplemental Materials (PDF) [file JCB_201610113_sm.pdf]

Supplemental material

JCB

Carroll et al., https://doi.org/10.1083/jcb.201610113

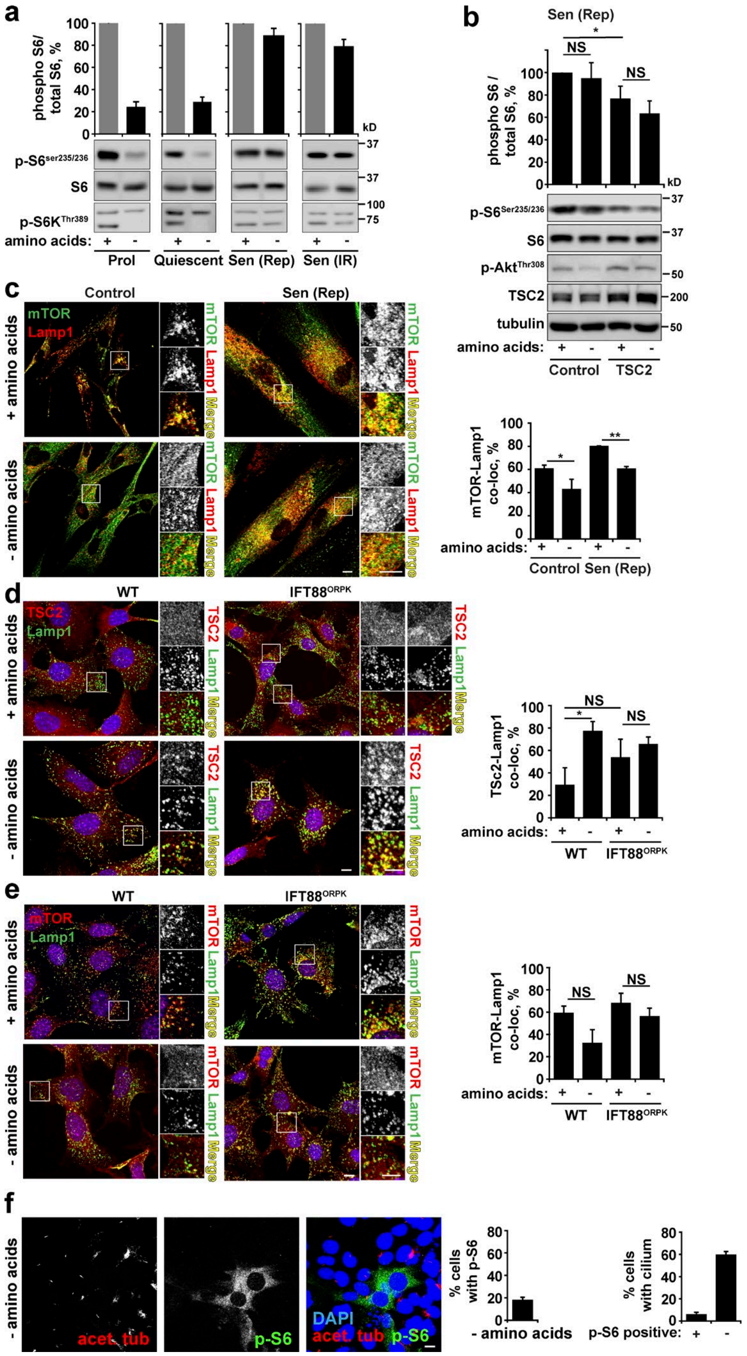

Figure S1. **Resistance of PI3K/Akt/mTOR signaling occurs in senescent and cilia-deficient cells, possibly via perturbation of mTOR localization.** (a) Primary fibroblasts were cultured in subconfluent (proliferating, Prol.) or confluent (quiescent) conditions or senescence was induced via replicative exhaustion (Sen (Rep)) or irradiation with 20 Gy (Sen (IR)). Cells were serum-starved overnight and then subjected to amino acid starvation for 1 h. Western blot analysis was performed for mTORC1 substrates phospho-S6K and phospho-S6 and quantified. (b) Sen(IR) cells were transduced with control or WT TSC2. Cells were maintained for a further 3 d after transduction before being incubated in the presence or absence of amino acids. Cells were lysed and immunoblotted. Note that the viral constructs are not tagged; therefore, the increased protein expression of TSC2 was monitored with anti-TSC2 antibody. (c) Proliferating or replicative senescent (Sen(Rep)) cells were treated as in panel a, fixed, and immunostained for antibodies against mTOR and Lamp1. Colocalization between Lamp1 and mTOR was analyzed. (d and e) WT and IFT88^{ORPK} chondrocytes were grown until confluent and subjected to serum starvation overnight with or without 1 h of amino acid starvation. Cells were fixed and immunostained with antibodies against TSC2 (d) or mTOR (e) and Lamp1. Colocalization was quantified. (f) Confluent HeLa cells were serum-starved overnight and starved of amino acids for 1 h. Cells were fixed and immunostained for acetylated tubulin and phospho-S6. The percentage of cells with phosphorylated S6 signal after starvation was quantified, as well as the percentage of that population (vs. the fully starved, phospho-S6–negative population) that formed cilia. S1f, *n* = 2 (error bars represent standard deviation); all others, *n* = 3 (for colocalization, at least 10 cells imaged per experimental repeat, and in all other panels, error bars represent SEM). Student's *t* test performed between groups: *, P < 0.05; **, P < 0.01. Bars, 10 µm.

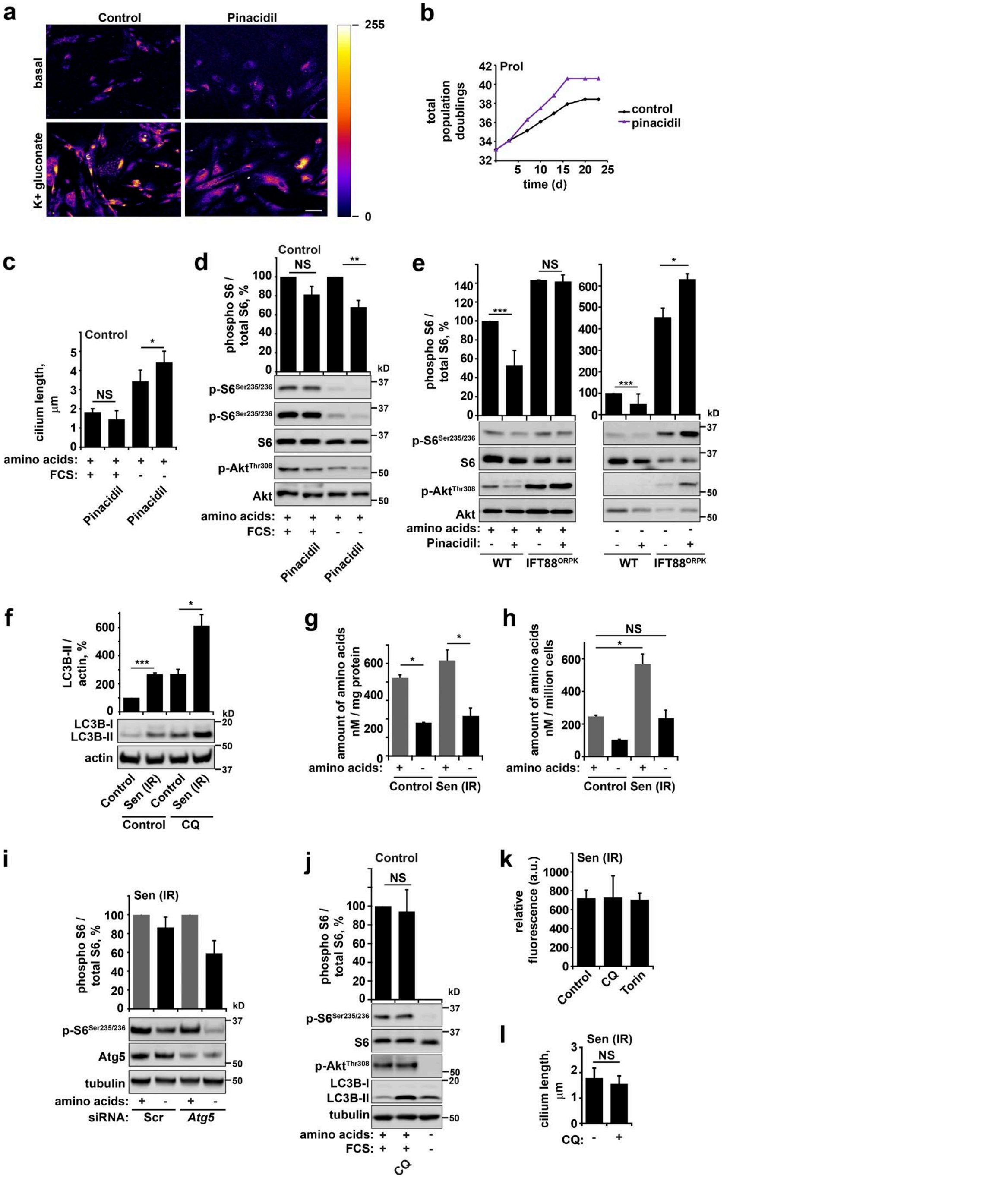

Figure S2. **Changes in plasma membrane potential and autophagy in senescence support persistent mTORC1 signaling.** (a) Representative images of potential-sensitive fluorescent dye, DiSBAC$_2$(3), showing that pinacidil treatment increases membrane potential of senescent fibroblasts. (b) Proliferating (Prol) primary fibroblasts were cultured in the absence or presence of pinacidil, and the population doublings were monitored over time. (c and d) Control fibroblasts were treated with 100 μM pinacidil overnight in the presence or absence of serum. Cells were fixed, stained for acetylated tubulin, and cilium length was quantified (c), or cells were lysed and immunoblotted to measure Akt and S6 phosphorylation (d). (e) Wild-type and IFT88^{ORPK} chondrocytes were grown until confluent and treated with 100 μM pinacidil overnight in the absence of serum. Cells were either lysed or subjected to 1-h amino acid starvation before lysis. Cells were immunoblotted to measure Akt and S6 phosphorylation. (f) Control and senescent (20 Gy irradiation; Sen (IR)) fibroblasts were incubated in full nutrient medium in the presence or absence of 50 μM chloroquine (CQ) for 2 h to block autophagosome degradation. Cells were lysed and immunoblotted to measure autophagy flux. (g and h) Control and senescent fibroblasts were serum-starved overnight and subjected to 1-h amino acid starvation. Cells were lysed and analyzed by LC-MS to measure intracellular levels of amino acids. Amino acid concentrations are represented normalized to protein levels (g) or cell number (h) because senescent cells are much larger than proliferating cells. (i) Senescent fibroblasts were treated with control (scramble, Scr) siRNA or siRNA against *Atg5* for 96 h. Cells were serum-starved overnight and starved of amino acids for 1 h. Cells were lysed and immunoblotted for mTORC1 activity. (j) Control fibroblasts were treated with 50 μM CQ for 1 h in the presence of amino acids and serum or completely starved. Cells were lysed and subject to immunoblotting. (k) Senescent cells were incubated with 50 μM CQ or 200 nM mTORC1 inhibitor/autophagy activator, Torin1, for 1 h. Membrane potential was measure as in panel a. (l) Senescent cells were serum starved overnight and incubated with 50 μM CQ for 1 h before cells were fixed and stained with antibodies against acetylated tubulin. Cilia length was quantified. Bar, 50 μm. Error bars represent SEM; a, b, and i, *n* = 2; all other experiments, *n* = 3. Student's *t* test performed between groups: *, $P < 0.05$; **, $P < 0.01$; ***, $P < 0.001$; NS, not significant.

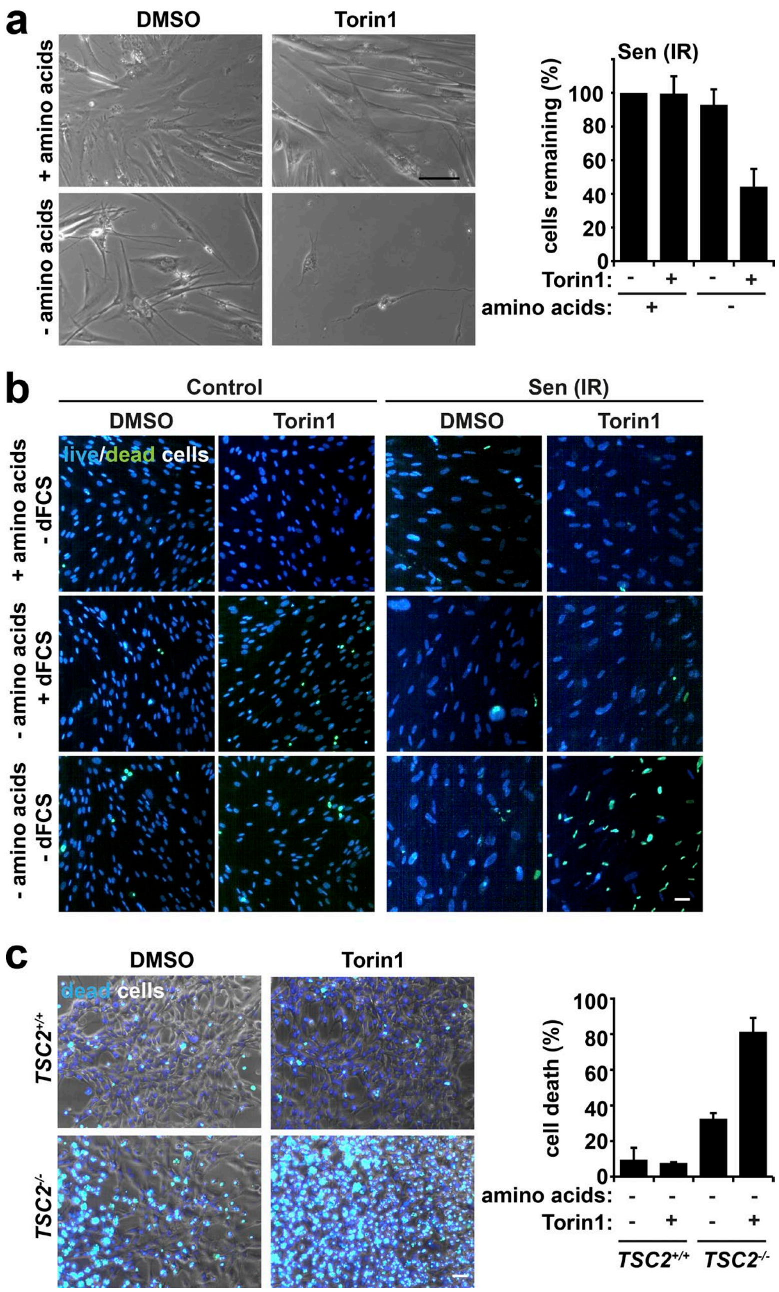

Figure S3. **Targeting persistent mTORC1 signaling to promote senescent cell death.** (a) Control or senescent (20 Gy irradiation (Sen(IR)) fibroblasts were starved of serum and amino acids in the presence or absence of 200 nM Torin1 for 24 h. Cells were imaged, washed once in PBS, and reimaged. The percentage of cells remaining per field of view was analyzed. (b) Senescent cells were treated as in a before being incubated with fluorescent ReadyProbe Cell Viability reagents. (c) *TSC2*$^{+/+}$ and *TSC2*$^{-/-}$ MEFs (which have persistent mTORC1 activation; Carroll et al., 2016) were starved of serum and amino acids in the presence or absence of Torin1 for 5 h and incubated with ReadyProbe Cell Viability reagents. Percentage of cell death was analyzed. Error bars represent SD; $n = 2$. Bars, 30 µm.

Reference

Carroll, B., D. Maetzel, O.D. Maddocks, G. Otten, M. Ratcliff, G.R. Smith, E.A. Dunlop, J.F. Passos, O.R. Davies, R. Jaenisch, et al. 2016. Control of TSC2-Rheb signaling axis by arginine regulates mTORC1 activity. *eLife*. 5:e11058. http://dx.doi.org/10.7554/eLife.11058
